# Respiratory Mononuclear Phagocytes in Human Influenza A Virus Infection: Their Role in Immune Protection and As Targets of the Virus

**DOI:** 10.3389/fimmu.2018.01521

**Published:** 2018-07-03

**Authors:** Sindhu Vangeti, Meng Yu, Anna Smed-Sörensen

**Affiliations:** Division of Immunology and Allergy, Department of Medicine Solna, Karolinska Institutet, Karolinska University Hospital Solna, Stockholm, Sweden

**Keywords:** emerging, virus, influenza, respiratory, monocyte, dendritic cell, macrophage

## Abstract

Emerging viruses have become increasingly important with recurrent epidemics. Influenza A virus (IAV), a respiratory virus displaying continuous re-emergence, contributes significantly to global morbidity and mortality, especially in young children, immunocompromised, and elderly people. IAV infection is typically confined to the airways and the virus replicates in respiratory epithelial cells but can also infect resident immune cells. Clearance of infection requires virus-specific adaptive immune responses that depend on early and efficient innate immune responses against IAV. Mononuclear phagocytes (MNPs), comprising monocytes, dendritic cells, and macrophages, have common but also unique features. In addition to being professional antigen-presenting cells, MNPs mediate leukocyte recruitment, sense and phagocytose pathogens, regulate inflammation, and shape immune responses. The immune protection mediated by MNPs can be compromised during IAV infection when the cells are also targeted by the virus, leading to impaired cytokine responses and altered interactions with other immune cells. Furthermore, it is becoming increasingly clear that immune cells differ depending on their anatomical location and that it is important to study them where they are expected to exert their function. Defining tissue-resident MNP distribution, phenotype, and function during acute and convalescent human IAV infection can offer valuable insights into understanding how MNPs maintain the fine balance required to protect against infections that the cells are themselves susceptible to. In this review, we delineate the role of MNPs in the human respiratory tract during IAV infection both in mediating immune protection and as targets of the virus.

## Introduction

Emerging viruses including influenza viruses, contribute significantly to human morbidity and mortality. Influenza is one of the oldest diseases known to mankind, with historical reports of influenza outbreaks dating as far back as 1173 (1). Still, influenza viruses are considered emerging/re-emerging viruses due to their capacity to dramatically change and cause epidemics with high mortality rate (2–5).

There are two forms of influenza: seasonal and pandemic. Seasonal influenza epidemics are caused by influenza A and B viruses and seasonal strains undergo mutations referred to as antigenic drift. For influenza A viruses (IAVs) antigenic drift is typically more pronounced each season, while it is more gradual for influenza B (6–9). Seasonal influenza epidemics contribute heavily to global disease burden and to deaths associated with lower respiratory tract (LRT) infections. 3–5 million cases of severe illness and 290–650,000 deaths annually are estimated, especially in young children, immunocompromised, and elderly people (10–13). The clinical picture of IAV infection is broad, ranging from mild/no symptoms, to viral pneumonia, severe respiratory failure, or acute respiratory distress syndrome. IAV infection results in increased susceptibility to secondary bacterial infections, which also contribute to mortality (14–16). In addition, circulating IAV strains can, at unpredictable intervals, cause influenza pandemics when the virus undergoes more dramatic genetic changes known as antigenic shift. Four pandemics have occurred in the past century: the 1918 Spanish flu, the 1957 Asian flu, the 1968 Hong Kong flu, and the 2009 Swine flu. Influenza pandemics are usually characterized by higher mortality than seasonal epidemics, often in age groups that are not typically at risk for influenza infections (17–23).

The nature and severity of influenza disease are influenced by the properties of the virus, host genetics, pre-existing immunity, and the immune response generated to varying extents—their relative contributions remaining incompletely understood (24–30). Highly pathogenic strains, like the Spanish flu, induce massive immune responses, suggesting that too potent antiviral immune responses are pathogenic rather than protective and that immunopathology is central in influenza (19, 31–37). Still, robust immune responses against IAV are required to control and clear infection (38–40). Mononuclear phagocytes (MNPs)— monocytes, dendritic cells (DCs), and macrophages (Mϕ)—are important in IAV infection as they are capable of limiting virus release; sensing and phagocytosing pathogens; clearing virus and apoptotic cells; releasing cytokines to mediate inflammation; directing leukocyte traffic *via* chemokine release; processing and presenting viral antigens; and finally activating naïve T cells (41–49).

The distribution and function of immune cells, including MNPs, differ between anatomical compartments (50–53). However, the exact nature of MNP involvement in human IAV infection remains largely unclear. Sampling the human respiratory tract in patients during ongoing infection poses significant challenges of accessibility and risk of causing further injury to the mucosal barrier. Defining human respiratory MNP distribution, phenotype, and function during IAV infection can therefore offer valuable insights into understanding how the immune system maintains the fine balance required to protect against infections. In this review, we will summarize insights on the role of MNPs in the human respiratory tract during IAV infection both in mediating immune protection and as targets of the virus.

## Human Respiratory MNPs

**Figure 1:**
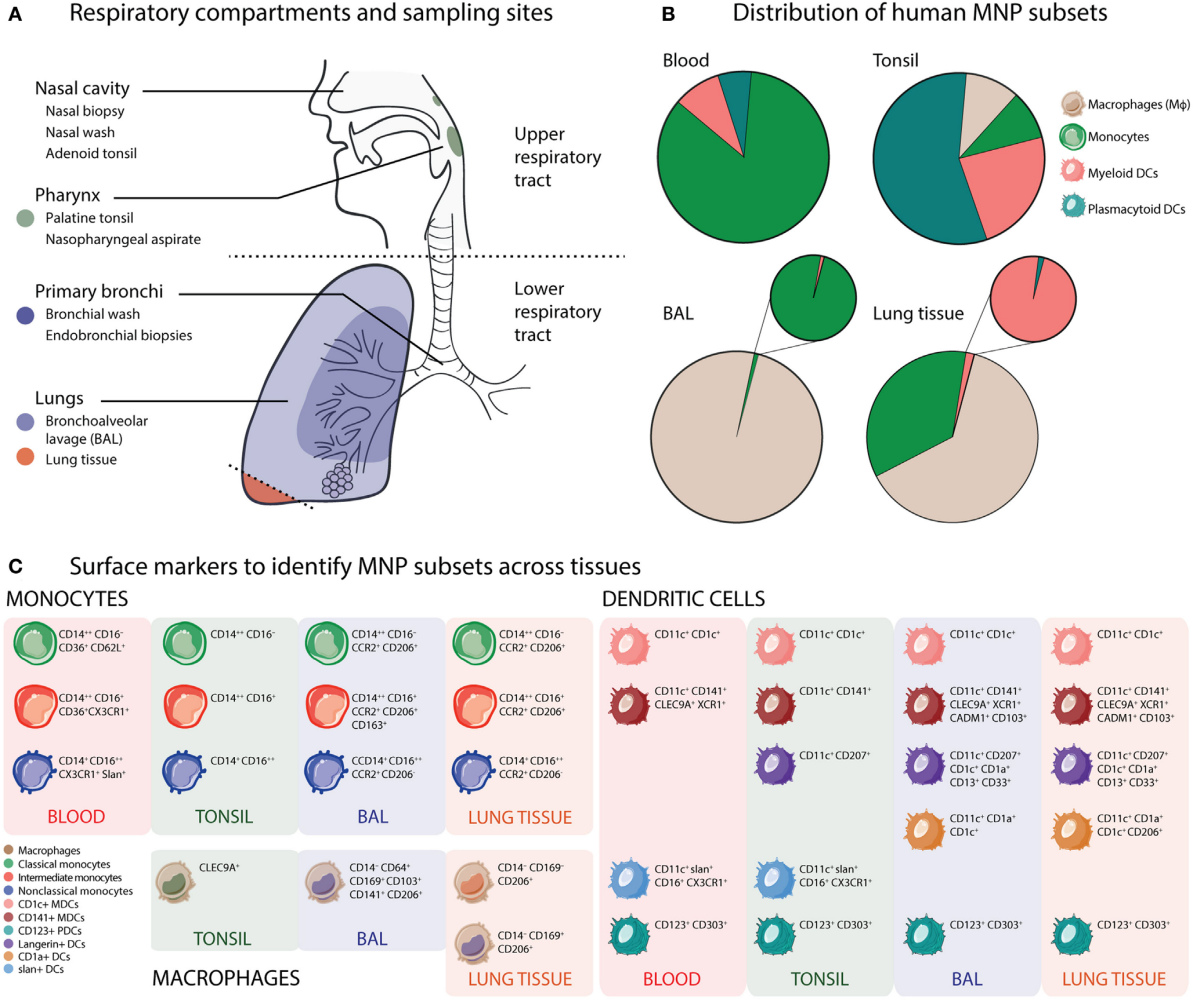
Mononuclear phagocyte (MNP) phenotype and distribution vary across human respiratory compartments. **(A)** Respiratory compartments and sampling sites. In the human upper respiratory tract, the initial site of influenza A virus infection, immune cells including macrophage (Mϕ), monocyte, and dendritic cell (DC) subsets from the nasal cavity and sinuses can be collected with nasal biopsies or nasal wash sampling. Along with pharyngeal palatine tonsils (and tubal and lingual tonsils), the adenoids form the Waldeyer’s ring, an anatomical structure comprising a ring of lymphoid tissue guarding the pharynx. In the lower respiratory tract, bronchoscopy allows sampling of discrete regions of the airways and lungs. Bronchial washes can be used to sample the cells lining the bronchi and bronchioles. Endobronchial biopsies can also be obtained from the mucosal tissue of the bronchial walls. Bronchoalveolar lavages (BALs) sample the most distal airways and alveolar sacs. Finally, lung resection samples allow sampling of lung parenchyma and tissue-resident immune cells. **(B)** Distribution of human MNP subsets. Pie charts illustrate broadly pooled data from 21 published studies on human MNP subset distribution in blood, tonsils, BAL, and lung tissue to demonstrate the differential distribution of MNPs across anatomical compartments reported from many research groups (51, 52, 57–61, 63–76). As different studies utilize different strategies to specifically define MNPs, the pie charts show groups of cells typically including several subsets of cells: Mϕs (beige), monocytes (green), myeloid DCs (MDCs) (coral), and plasmacytoid DCs (PDCs) (teal). **(C)** Surface markers to identify MNP subsets across human tissues. The various MNP subsets across tissues can be identified using flow cytometry from HLA-DR+ leukocytes that do not express lineage (T cells, B cells, NK cells, and granulocytes) markers. Apart from CD123+ PDCs, the MNP subsets express different levels of the myeloid marker CD11c. Mϕs have been studied in detail in both BAL and lung tissue, where CD169 expression distinguished alveolar from interstitial Mϕs. Monocyte subsets can be identified from most tissues based on relative expression of CD14 and CD16, as first defined in blood. The major MDC subsets are defined by expression of CD1c or CD141. The extended MDC subsets are now distinguished by expression of CD207 (langerin), CD1a, or slan (51, 52, 57–61, 63–76).

The human respiratory tract encompasses a large mucosal surface with the densest vasculature of all organ systems, that is constantly exposed to the external environment with every inhalation (54, 55). MNPs are positioned along the respiratory tree, in anticipation of exposure to foreign material and respiratory pathogens. MNPs are dually tasked with both promoting inflammation and maintaining tolerance, without disrupting the mucosal barrier that separates the air-filled alveolar spaces from sterile blood in the capillaries (56). A detailed understanding of the distribution and function of respiratory MNPs from the nasal cavities to the alveoli is essential, yet currently incomplete, largely due to the challenges of accessing these tissues in humans. However, recent studies have generated important insight in this area and Mϕs, monocytes, monocyte-derived DCs (mo-DCs), and bona fide DC subsets have been identified from healthy human respiratory tissues. Figure 1 summarizes the current understanding of the phenotype and distribution of human MNP subsets in respiratory tissues at steady state, as reported (41, 51, 52, 57–62).

Alveolar macrophages (AMϕs) are the most abundant phagocytes of the human lungs, responsible for internalizing inhaled pathogens and antigens, and comprising 95% of cells sampled *via* bronchoalveolar lavage (BAL) (51, 58, 60, 77). Interstitial macrophages, a functionally distinct population of Mϕs residing in lung parenchymal tissue, are less accessible and thus less well studied (63, 78). Similar to monocytes in blood, respiratory monocytes have been characterized as classical monocytes (CMs: CD14+ CD16−), intermediate monocytes (IM: CD14+ CD16+), and non-classical monocytes (NCMs: CD14− CD16+) (51, 52, 58, 64–66). IMs are more frequent in the airways, as opposed to blood, where CMs are in abundance; while NCMs seem to be the rarest monocyte subset (51, 58–60). CMs are the first cells to migrate out of blood to infiltrate sites of inflammation, release chemokines to attract other leukocytes; and can differentiate into mo-DCs and Mϕs (67, 68). IMs represent a population of differentiating monocytes that have been reported to expand during inflammation and/or infection (79–82). NCMs have been attributed with patrolling functions, debris removal, promoting wound healing (64, 81), and to some extent, TLR3 mediated type I interferon (IFN) production (69). Mo-DCs are an interesting subset that transiently arises in tissues from (primarily classical) monocytes recruited to the site of inflammation (46). In comparison to monocytes, DCs are rare in blood, and rarer still in the airways. Subsets of CD11c-expressing myeloid DCs (MDCs); CD1c+ MDCs, CD141+ MDCs, and more recently, langerin+ MDCs (with variable CD1a expression), as well as CD123+ plasmacytoid DCs (PDCs) have been described in the human respiratory tract (51, 57–60, 70–72). MDCs are excellent antigen-presenting cells, CD141+ MDCs specialize in cross presentation *via* MHC I; and PDCs excel at type I IFN-mediated antiviral protection.

In the human respiratory system, the upper respiratory tract (URT) is comprised of the nasal cavity, sinuses, and the pharynx (Figure 1A). The LRT including the trachea, bronchi, bronchioles, and alveoli, is typically divided into the proximal conducting zone and the distal respiratory zone (Figure 1A) (83). The LRT accounts for a larger cumulative surface area and consequently higher likelihood of pathogen–immune cell interactions. However, it is the URT that is initially involved in prevention of pathogen entry (83). MNP distribution in the URT, especially at steady state, also remains poorly characterized. Recent studies have shown Mϕs, CMs, MDCs, and PDCs in the nasal cavities (84, 85); CMs in the sinuses; CMs, MDCs, and PDCs in the nasopharynx (43, 44); CD1c+ MDCs in nasal tissue (86); and Mϕs, CMs, and several DC subsets (PDC, CD1c+, CD141+, CD207+, slan+, Axl+, and CD4+) have been described in human tonsils (73–76). What is evident, however, is that the relative distribution of MNP subsets at steady state varies greatly across the different compartments of the respiratory tract (51, 87). For example, in blood, monocytes greatly outnumber all other MNP subsets, whereas in tonsils, PDCs are the most abundant MNP subset. In BAL, AMϕs make up almost 95% of all cells, but IMs are more frequent than DCs. In lung tissues, both alveolar and interstitial Mϕs can be found at different frequencies. Monocytes and MDCs are also present at greater frequencies than PDCs (Figure 1B). The immunological map of the human respiratory tree is becoming more detailed (Figure 1C), enabling a better understanding of how the respiratory immune system changes during disease including respiratory viral infections like IAV.

## MNPs: Innate Immune Responders in IAV Infection

Respiratory MNPs function as mucosal sentinels and come into play rapidly after onset of IAV infection. Monocytes and DCs resident in the nasopharyngeal mucosa can rapidly sense the presence of IAV and elicit an early response featuring a predominance of monocyte-recruiting chemokines like CCL2, CCL17, CX3CL1, and MCP3 (45, 88, 89). Mϕs, that are abundant in the LRT, are less likely to be involved in uncomplicated human IAV infections, when the virus typically remains localized in the URT. However, when the virus spreads lower toward the lungs, not uncommon among pandemic IAV strains, Mϕs are likely central in the innate immune response.

**Figure 2:**
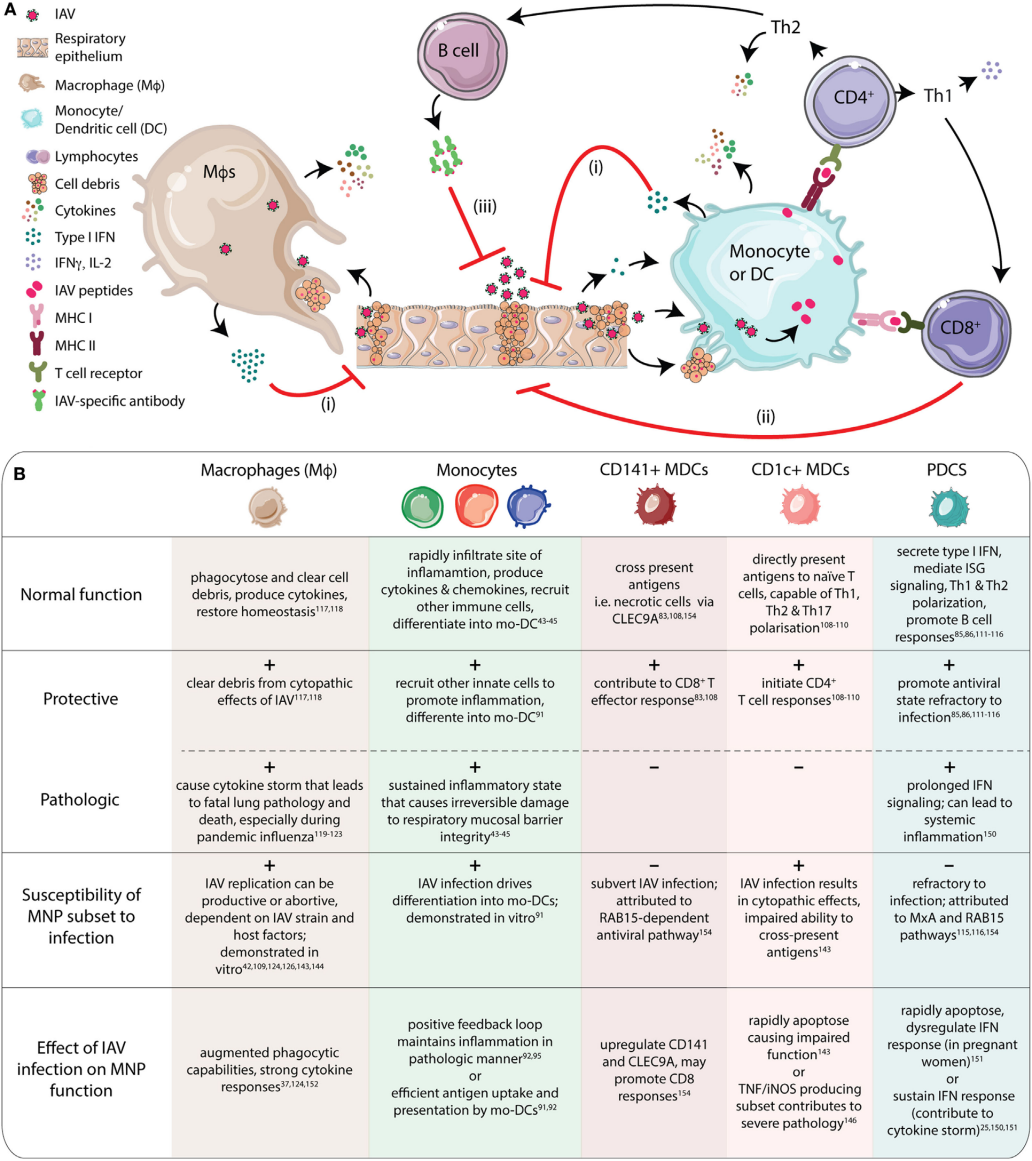
Human mononuclear phagocytes (MNPs) play a multitude of roles to mediate immune protection during influenza A virus (IAV) infection. **(A)** MNP subsets have many overlapping functions. Macrophages (Mϕs) clear up cell debris and release cytokines. Monocytes and dendritic cells (DCs) can also release cytokines and present antigens to initiate adaptive responses. (i) Following IAV infection of respiratory epithelium, Mϕs, monocytes, and DCs respond to the virus and cell debris, launching potent cytokine responses (TNFα, IL-6, IL-12p40, and IL-10), including interferon (IFN)α. Induction of interferon-stimulated genes (ISGs) promotes an antiviral state in bystander cells, protecting them from infection. (ii) The antigens taken up by monocytes/DCs are processed and presented *via* MHC I and II to CD8+ and CD4+ T cells, respectively. Antigen-specific CD8+ T cells perform effector functions *via* cytotoxic granule- and FasL-mediated caspase-dependent apoptosis. (iii) CD4+ T cells mature into subsets with specific functions. Th1 cells primarily produce IFNγ, IL-2, and TNFβ; and aid CD8+ T cell proliferation. Th2 cells on the other hand, produce IL-4, IL-5, and IL-13 and assist B cells, especially during antibody class switching, promoting production of neutralizing antibodies. Induction of broadly neutralizing antibodies against all strains of influenza virus remains a challenge in the field of influenza immunology (45, 57, 75, 76, 96–106). **(B)** The table summarizes the individual functions of MNP subsets that can protect against IAV infection, but also contribute to pathology. Most MNP subsets are susceptible to IAV infection, as demonstrated by *in vitro* studies. As a consequence of IAV infection, MNP function can be directly affected, prompting them to respond in a protective or pathologic fashion (25, 37, 42–45, 73, 75, 76, 91, 102–105, 107–127).

The diverse functional capacity of monocytes translates into their involvement in several aspects of immunity to IAV, as depicted in Figure 2. Monocytes rapidly infiltrate the URT following IAV infection where increased nasal CM numbers and cytokine (MCP3, IFNα2, and CCL17) levels can predict disease severity (43–45). Similarly, in patients infected with the pandemic A/CA/07/09 (pH1N1) strain, high numbers of CD14+, TNF-producing monocytes were reported in blood, that positively correlated with disease severity in young, otherwise healthy adults (88, 90). In addition, exposure to IAV also drives differentiation of monocytes into mo-DCs *in vitro* (91). Studies on human IAV infections demonstrate causal association between CCR2-dependent lung monocyte and mo-DC recruitment and IAV-induced mortality in an NOS-2-dependent manner (91–95).

Mucosal tissue-resident DCs in peripheral tissues like the respiratory tract sense and take up antigens. They then migrate to draining lymph nodes to present processed antigen to T cells. Antigen-specific, clonally expanded T cells migrate back to the site of infection to control and clear infection (128–131) (Figure 2). This process is critical to restoration of homeostasis as well as for induction of potent adaptive immune responses. Murine models have elegantly demonstrated DC function during IAV infection (96–100, 132). What remains to be described is the exact role of human DC subsets. During pediatric IAV infection, MDCs and PDCs mobilize to the nasopharynx while DC numbers are reduced in blood (43, 44). The potential redistribution of DC subsets remains to be characterized in adults as well as over the course of infection. The different DC subsets each likely perform individualized tasks during IAV infection. CD1c+ MDCs are the most abundant MDCs in the airways (51, 58, 60), and are excellent at pathogen recognition (101, 133), inducing expansive T helper responses (107–109); and cytokine secretion (101). CD141+ MDCs possess superior MHC I cross-presenting abilities that can aid IAV clearance by CD8+ T cells (73, 107). TLR3 mediated cytokine production (TNF, IL-6, IL-12, and IFN-β) and importantly, type III IFN production by CD141+ MDCs, assist in enhanced innate MNP protection against IAV (57). PDCs mediate type I IFN-dependent antiviral protection that is beneficial during IAV infection. In addition to transcriptional activation of many IFN-stimulated genes (ISGs), PDCs also promote both T and B cell responses (75, 76, 102–104, 110–112).

Macrophages contribute during IAV infection by clearing cell debris, chemokine and cytokine production to modulate inflammation, recruitment of other MNPs, and to restore subsequent tissue homeostasis (Figure 2A) (105, 113). AMϕs are of particular importance when the infection reaches the LRT, where the AMϕs are in vast abundance. Severe influenza with LRT pathology is often accompanied by AMϕ involvement (114–118). Unhindered AMϕ-associated cytokinemia can result in devastating consequences for patients, ranging from delayed recovery to fatal lung pathology (116). Several factors control the extent of Mϕ involvement, two of the most likely contributors being IAV subtype/strain and Mϕ phenotype (90, 105, 113, 114, 118–120, 134) (Figure 1C). For example, Mϕ cytokine production differs across H5N1 and pandemic/seasonal H1N1 strains (119). The protective and pathologic roles of MNP subsets during IAV infection have also been summarized in Figure 2B.

Murine models of IAV infection have extensively characterized the role of MNPs in antiviral protection (92, 100, 132, 135–137). A potent immune response to human IAV infection is also likely dependent on synergy between the different MNP subsets and their functions (53, 138–141). However, MNP susceptibility to IAV infection can easily upset the balance, impacting both virus clearance and return to homeostasis.

## MNPs and Respiratory Epithelium (RE): Mucosal Barriers and Targets of IAV Infection

During IAV infection, the virus is largely confined to the airways, where the RE is primarily targeted (13, 106, 142–147). The RE and MNPs represent an interesting functional dichotomy—both are targets of the virus and also capable of immune functions to limit infection (25). The epithelial tight junctions constitute a mechanical barrier against the exterior and secrete antiviral molecules. The RE senses IAV *via* TLRs and RIG-I; with RIG-I signaling concentrated at the tight junctions, resulting in type I and type III interferon-mediated antiviral protection (106). Chemokines secreted from the RE aid neutrophil and MNP recruitment to the site of infection, enhancing innate protection. While potently responding to IAV, the RE is also highly susceptible to the cytopathic effects of IAV infection (Figure 2A). Loss of mucosal barrier integrity promotes bacterial adherence, contributing to secondary bacterial infections and lung pathology often associated with severe IAV infection (118, 147–149).

Mononuclear phagocytes are well located in the human respiratory mucosa to be targeted by the virus upon entry (135), and the endocytic and migratory properties of MNPs are likely favorable to viral infection and dissemination (120, 134). *In vitro* IAV infection of human Mϕs and DCs has been shown to result in productive infection with release of infectious particles (119–121) but has also been reported to result in abortive infection (42, 108, 121, 122), the contrast being discussed in great detail in Ref. (42). Which of these alternatives prevail in clinical cases, and what host factors determine their own fate, are questions that are yet to be answered. In addition, the negative implications of IAV infection, from an immunological perspective, they may be more pronounced for MNPs than for epithelial cells as MNPs are central in establishing a protective, specific immune response.

## Consequences of IAV Infection of MNPs

Mononuclear phagocyte susceptibility to IAV infection can impair their many functions. For example, MDCs are crucial for T cell activation but they are also readily susceptible to IAV infection, impairing their ability to present antigens *via* both the direct presentation and cross presentation pathways (46, 121, 150). Most seasonal and low-pathogenic IAV strains infect respiratory human Mϕs and DCs but replication is typically abortive and therefore skews in favor of host defense (120). However, highly pathogenic strains of IAV can overcome this barrier and productively infect Mϕs and DCs, which in turn can impact viral amplification, dissemination, as well as pathogenicity and immunogenicity (123). Primary human monocytes exposed to H5N1 or highly pathogenic avian influenza strains *in vitro* exhibit a reduced antiviral response, as a consequence of impaired NF-κB signaling (91, 114, 115). In a murine model of IAV infection, CCR2+ inflammatory monocytes accumulate in lungs (92, 94). Impaired virus clearance by MNPs triggers IFN-mediated recruitment of CCR2+ monocytes inflammatory in a positive-feedback loop, resulting in severe lung pathology (92) (Figure 2B).

Impaired MNP responses have also been observed in IAV patients. Peripheral blood monocytes and to some extent PDCs, exhibit attenuated IFN responses indicating dysregulation at a systemic level, in particular in infants and the elderly, two of the largest risk groups for severe influenza disease (151–153). Human PDCs that potently produce large amounts of type I IFN, in response even to low doses of IAV, can rapidly undergo apoptosis when exposed to high doses of the virus (25, 124). Possibly related to that, it has been reported that pregnant women, a risk group for influenza, have fewer PDCs in circulation that are also less efficient at IFN production, which could contribute to more severe IAV disease during pregnancy (125) (Figure 2B).

As undesirable as depressed MNP function is, excessive activation of MNPs can also be equally dangerous, by contributing to IAV-induced immune pathology leading to fatal respiratory distress. Human monocyte-derived pro-inflammatory Mϕs exposed to IAV *in vitro* exhibit augmented phagocytic capability and strong cytokine responses (119). While this can encourage adaptive responses, it also contributes to the cytokine storm that is a hallmark of severe influenza disease (37, 126). Prolonged IFN signaling can also destroy alveolar epithelium and contribute to development of secondary bacterial infections, the most common complication associated with influenza infections (93). TNF/iNOS-producing DCs, a subset of inflammatory DCs, accumulate in the LRT and promote CD8+ T cell responses in an IAV mouse model, but are also positively correlated with higher lethality (123). However, *in vitro*, human CD8+ T cells can rapidly induce monocyte differentiation into tip-DCs that in turn prime naïve CD4+ T cells and promote protective Th1 responses (154) (Figure 2B).

Not all respiratory MNP–IAV interactions have adverse implications. Virus-induced human *in vitro* mo-DCs express both CLEC9A and CD141, as do blood CD141+ MDCs. But uniquely, mo-DCs express CD141 on the cell surface and CLEC9A intracellularly (91). CD141+ DCs can efficiently prime and drive CD8+ T cell proliferation, while CLEC9A is linked to antigen uptake. CD141+ MDCs also subvert IAV infection by resisting virus entry in a RAB-15 dependent manner, instead relying on uptake of apoptotic virus-infected CD1c+ MDCs (and other cells) as a source of antigens (127) (Figure 2B). Virus-induced CD141+ DCs also exhibit type I IFN secretion and upregulate ISGs (tetherin, viperin, and IFITM3) and RIG-I/MDA5, suggesting an important protective role for them during infection; despite poor expression of co-stimulatory molecules (CD40, CD86, and HLA-DR), weaker pro-inflammatory cytokine expression, and impaired ability to activate naïve CD4+ T cells (46). Induction of CD141+ DCs could therefore be employed in vaccination/therapeutic strategies. To summarize, while IAV infection of MNP compromises some aspects of innate protection, biological redundancy due to the overlapping functions of MNP subsets can likely prevent loss of essential immune responses.

## Concluding Remarks

Respiratory MNPs are important in the immune responses to IAV infection. At the same time, MNP susceptibility to IAV infection poses an interesting immunological challenge. Several key questions still remain to be further addressed to understand this dichotomy better. Does compromised MNP function result in altered innate immune responses? Do altered innate immune responses subsequently impair efficient induction of adaptive responses, ultimately contributing to increased host morbidity and mortality? If on the other hand, robust, unchecked innate responses lead to prolonged inflammation, causing irreparable damage to the host, is there a commonality in host responses across the various demographics affected by influenza? To answer these questions, and delineate the role of respiratory MNPs in human IAV infection, it will be critical to detail the function of the different MNP subsets—for example, functional assessment of sorted cells from the respiratory system and performing RNA sequencing or epigenetic analyses. Prospective studies of human IAV patients where detailed analyses of tissue samples can be correlated to clinical parameters are likely required to fully understand how MNPs contribute to disease severity.

## Author Contributions

SV and MY performed the literature review. SV designed the figures. SV, MY, and AS-S organized and wrote the manuscript. SV and AS-S edited the manuscript.

## Conflict of Interest Statement

The authors declare that the research was conducted in the absence of any commercial or financial relationships that could be construed as a potential conflict of interest. The handling Editor declared a shared affiliation, though no other collaboration, with the authors.

## Abbreviations

AMϕ, alveolar macrophage; ARDS, acute respiratory distress syndrome; BAL, bronchoalveolar lavage; CM, classical monocyte; DC, dendritic cell; IAV, influenza A virus; IFN, interferon; IM, intermediate monocyte; IMϕ, interstitial macrophage; ISG, interferon-stimulated gene; LRT, lower respiratory tract; MDC, myeloid dendritic cell; MNP, mononuclear phagocyte; Mo-DC, monocyte-derived dendritic cell; Mϕ, macrophage; NCM, non-classical monocyte; PDC, plasmacytoid dendritic cell; RE, respiratory epithelium; tipDC, TNF/iNOS-producing dendritic cell; URT, upper respiratory tract.
